# Sentinel Lymph Node Biopsy in Breast Cancer Patients by Means of Indocyanine Green Using the Karl Storz VITOM® Fluorescence Camera

**DOI:** 10.1155/2018/6251468

**Published:** 2018-03-26

**Authors:** Thomas Papathemelis, Evi Jablonski, Anton Scharl, Tanja Hauzenberger, Michael Gerken, Monika Klinkhammer-Schalke, Matthias Hipp, Sophia Scharl

**Affiliations:** ^1^Frauenklinik, Klinikum St. Marien Amberg, Amberg, Germany; ^2^Tumorzentrum der Universität Regensburg, Institut für Qualitätssicherung und Versorgungsforschung, Regensburg, Germany; ^3^Klinik für Strahlentherapie, Klinikum St. Marien Amberg, Amberg, Germany; ^4^Klinik für Radioonkologie und Strahlentherapie München, Technische Universität München, München, Germany

## Abstract

Currently, the use of radioisotope and blue dye for sentinel lymph node biopsy (SLNB) for axillary staging in breast cancer is common. Recently, indocyanine green (ICG) has been proposed as an alternative sentinel lymph node (SLN) tracking agent. We evaluated the clinical value of ICG as an additional tracer in combination with Technetium^99m^ and as an alternative to Technetium^99m^ for the identification of SLN in 104 breast cancer patients. 21 patients had at least 1 histologically tumor-positive SLN. All 21 patients were detected by ICG; in one of these 21 sentinel-positive patients, Technetium^99m^ was unable to identify lymph node involvement. Our results show that ICG is as effective as the radioisotope for SLNB. In addition, as a near-infrared dye, it has the advantages of real-time visualization, lower cost, and wider availability, since no radioactive material needs to be handled. This trial is registered with German Clinical Trial Register Main ID: DRKS00013606.

## 1. Introduction

Breast cancer has the highest death toll of all cancers in women worldwide [1]. Axillary lymph node involvement (nodal status) has been shown to be the most significant prognostic factor and is therefore routinely assessed during primary treatment. Early detection of breast cancer is correlated with a significantly improved prognosis and a significantly reduced percentage of patients with involved axillary lymph nodes (N+) [2]. In patients with clinically negative nodes (cN0), sentinel lymph node biopsy (SLNB) has comparable diagnostic accuracy for acquiring nodal status as axillary dissection with significantly reduced morbidity; therefore, it has become the standard procedure for axillary staging in cN0 breast cancer patients. Based on standardized and quality-assured implementation, SLNB shows a high staging accuracy (>90%) [3–8]. Standard SLNB procedures nowadays include periareolar or interstitial injection of radioactive Technetium^99m^ (Tc^99m^) colloid, blue dye, or a combination of both [7], whereupon in Germany radiocolloid is widely considered the gold standard [6].

The use of radioisotopes has proven the highest detection rates, yet it has several drawbacks such as limited availability, high costs, no real-time tracing, and exposure of patient and surgeon to radioactivity [9]. Blue dye is easy to handle and cost-effective, yet it has lower detection and higher false-negative rates, cannot be seen through skin or fatty tissues, and can cause allergic reactions in rare cases, even anaphylaxis [10]. The dual-tracer method using blue dye in addition to Tc^99m^ has been demonstrated to be superior to either agent used separately. However, this improvement lacks enough clinical significance for rating dual tracer as mandatory [7, 8, 11–13].

As a near-infrared (NIR) imaging agent, ICG can be traced in real time in high resolution, is cost-effective, and is broadly applicable [14]. Moreover, it has FDA and EMEA approval to be used for imaging blood flow and therefore may be used off-label as a lymphatic tracer in clinical trials. Recently indocyanine green (ICG) fluorescence has been suggested as an alternative dye for tracing SLN in breast cancer [15–19]. Several clinical trials and studies evaluating the use of ICG for SLNB have demonstrated promising results, showing higher detection rates as compared to blue dye and equal sensitivity as radioisotopes [17].

The present retrospective study adds more experience to assess the potential of ICG as a combination partner for dual-tracer detection together with TC^99m^ colloid or as an alternative to this current standard SLN detection technique.

## 2. Materials and Methods

### 2.1. Study Design

This study is a retrospective single-arm, single-center study. It was designed to assess whether the use of ICG fluorescence adds additional benefit to Tc^99m^ colloid in SLN detection in early breast cancer. As an additional endpoint, we wanted to learn the accuracy of ICG fluorescence as an alternative tracer method compared to the use of the radioactive colloids for SLNB. The Bavarian State Medical Association (Bayerische Landesärztekammer) confirmed that no review by the ethics committee is necessary for this retrospective study.

St. Marien-Hospital (Amberg, Germany) is certified as oncology center and breast care center by the German Cancer Society and German Society of Senology [20] and part of the Comprehensive Cancer Center Erlangen-EMN [21]. It is properly equipped and authorized to perform SLNB by means of radioisotopes (Technetium^99m^) as well as ICG fluorescence. Certification as breast care center requires that certified surgeons with proven and continuous experience in breast surgery perform the procedure. In accordance with these prerequisites, only 3 designated surgeons executed SLNB.

### 2.2. Patients and Procedures

Between June 2016 and May 2017, all patients with breast cancer who presented at the Breast Care Center of St. Marien Amberg (Amberg, Germany) and had an indication for SLNB were offered dual-tracer SLN detection using Tc^99m^ colloid and ICG. According to German guidelines, SLNB is suggested in all cN0 patients regardless of whether a breast-conserving surgery or a mastectomy is performed; in case of neoadjuvant systemic therapy (NAST), the procedure is preferably performed after its completion. We complied with these guidelines. Our patient collective included patients undergoing breast conserving surgery and mastectomy and also patients receiving SLNB after NAST. German guidelines consider radioactive colloid as standard and the use of dye in addition as optional but not required and ICG as a suitable dye [7, 8]. We therefore suggested ICG as a dye technique. Patients were informed that using dye in addition to Tc^99m^ is not mandatory and ICG use for SLNB is off-label and provided written informed consent. Patients with iodine or ICG allergy or those who did not consent were excluded from ICG use. This retrospective analysis includes all 104 patients who had dual labeling with Tc^99m^ and ICG within the above-mentioned time period.

SLN mapping was done according to the protocols recommended and used in the SENTINA study [22, 23] by periareolar injection of Technetium^99m^ radioactive colloids (50 MBq Tc-99m-Nanocoll) the day before surgery. In addition, 30 minutes prior to surgery, 0,5 ml of ICG solution (0.77 mM) was injected subcutaneously into the periareolar region at 3, 6, 9, and 12 o'clock. In total, 3.33 mg of ICG was injected, which is far below the maximum allowable dose of 5 mg/kg body weight; as such, no adverse reactions were to be expected.

**Figure 1:**
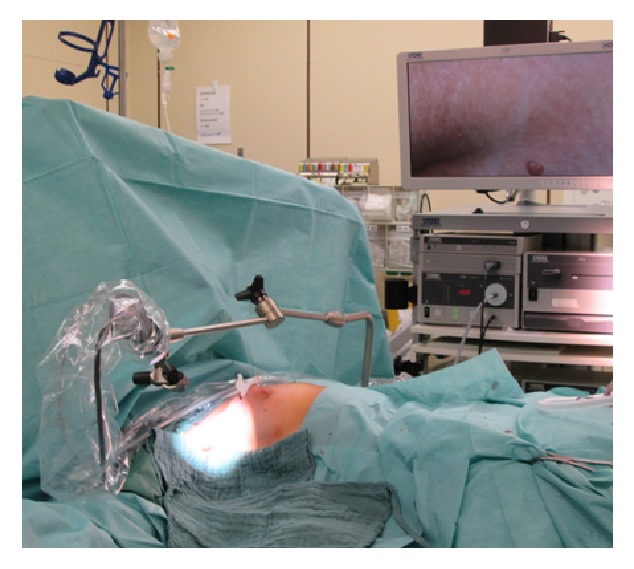
The Karl Storz VITOM HD System, an exoscope using extracorporeal magnification coupled with a high-definition fluorescence camera system. The exoscope is fixed by a holding arm attached to the operating table and connected to a HD monitor.

**Figure 2:**
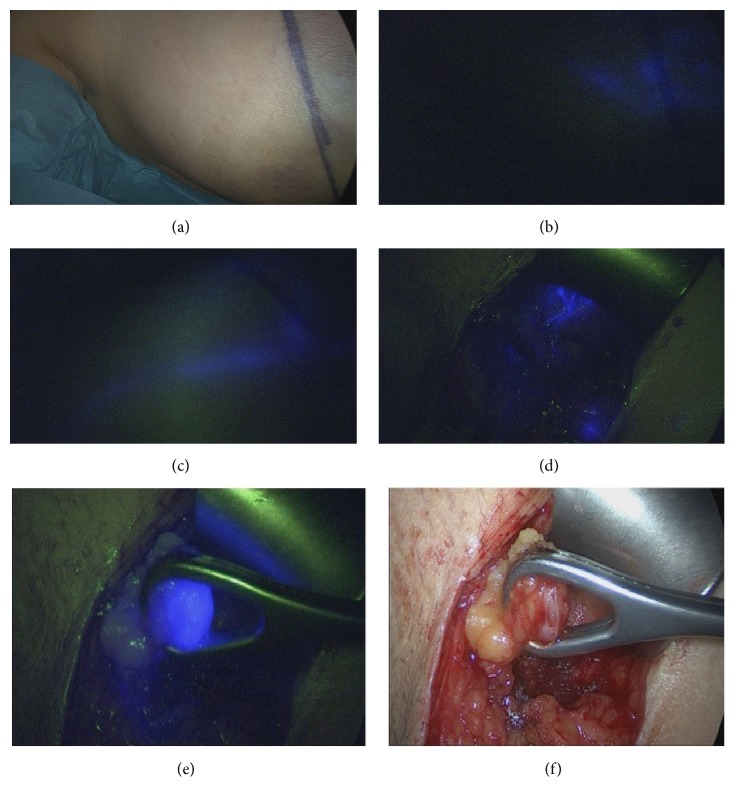
Sentinel lymph node mapping with ICG fluorescence using the Karl Storz VITOM camera. Breast with marked incision line for tumor-adapted oncoplastic reduction plasty in clear light (a) and after switching to NIR fluorescence (b), with the latter image displaying a fluorescent lymphatic vessel, which runs from the areola to the axillary region (c). After skin incision, fluorescent lymphatic vessels appear (d). The sentinel lymph node is presented by clear shining blue (e) and can be identified as lymph node in clear light (f).

SLNB was performed before breast surgery, which followed only after the axillary procedure was completed. The Karl Storz VITOM HD System, an exoscope using extracorporeal magnification coupled with a high-definition fluorescence camera system (Figure 1), was utilized to trace ICG. The operating room was darkened during axillary surgery. Switching from “normal” to near-infrared (NIR) light and back allowed visualizing anatomy and fluorescence, respectively. Lymphatic vessels containing ICG were visualized transcutaneously and followed to the axillary region and into the axilla through an approximately 4 cm longitudinal incision of the skin along the anterior axillary anatomical line. The transcutaneous visibility of the lymphatic vessels was helpful to determine where exactly the skin cut should be made but not required. In fact, lymph vessels were not seen transcutaneously in more patients than vice versa. In these cases, they were detected after skin incision in the anterior axillary anatomical line. Following these lymph vessels leads to the ICG positive nodes (Figure 2). In order to avoid leakage of ICG from the lymphatic system, which could discolor the surgical field and interfere with the color in lymph nodes, we tried not to cut the lymphatic vessels by merely spreading the tissue and not dissecting it. All ICG-positive nodes were sampled. Dissection was finished when no fluorescent lymph vessels guided the surgeon to additional nodes. Only after all real-time ICG-mapped SLN were harvested from the axilla did we assess whether they contained Technetium^99m^ activity using a Neoprobe Gamma Detection System. Then the axilla was checked for radioactivity and if a significant amount was present, Technetium^99m^-marked SNL was subsequently removed. Operation times for SLNB varied from 15 to 40 minutes and shortened with increasing experience with ICG.

All SLN were analyzed for the presence of tumor by histologic examination, the work-up following the recommendations of the German guidelines [7]. Because these guidelines allow omitting axillary dissection in certain cases with positive nodes [8, 20], the frozen section of lymph nodes lacks clinical consequences and was therefore not performed.

### 2.3. Statistical Analysis

The objective of this study was to assess the sensitivity of ICG fluorescence for the detection of SLN in addition to and as compared to the standard radioactive method with Technetium^99m^. All analyses were performed using IBM SPSS Statistics version 23.0 (Chicago, USA).

## 3. Results

### 3.1. Patient Tumor Characteristics

The average age of the 104 patients with dual-tracer SLNB was 59.4 (range: 27–84) and the average BMI was 26.8 (range: 20–39). 19 patients have been submitted to SLN mapping after NAST; 15 of them had complete histologic remission of the primary tumor (pCR), whereas the other 4 patients had residual tumor. No differences in terms of the ICG mapping procedure have been reported by the surgeons after NAST when compared to primary surgery. In 58 patients where surgery had been performed prior to systemic therapy, tumor sizes were smaller than 2 cm (56%). In 17 other patients, tumors measured between 2 and 5 cm (16%). In 3 patients, the tumor diameters were larger than 5 cm and in 2 cases the tumor had already reached the thoracic wall. The majority of patients underwent breast-conserving surgery (85 versus 19). No adverse reactions associated with ICG were noted.

Out of the 104 breast cancer cases, 5 have been excluded from further analysis because they had SNLB in spite of clinically suspicious nodes (cN1) and therefore did not fulfill the criteria for SLNB [6–8].

### 3.2. SLN Detection and Analysis

In total, 220 SLN were dissected (mean per patient: 2.2; range: 1–7) (Table 1); of those 215 (97.7%, 95% CI: 95.8–99.7%) were ICG fluorescent and 172 were positive for Technetium^99m^ (78.2%, 95% CI: 72.7–83.6%), respectively. 5 nodes (2.3%) were not visible by ICG but were detected through gamma probing of the axilla. On the other hand, 48 nodes (21.8%) were identified with ICG fluorescence only, showing no signal in gamma probing.

In all 99 patients included in the analysis, at least one SLN was detected by Technetium^99m^ and/or ICG, meaning that SLN detection rate by dual mapping was 100%. In 95 patients, at least one node was picked up by both tracers, ICG and Tc^99m^. In the 4 cases where no lymph node was marked with both tracers, in 2 women at least one node was detected by Tc^99m^ and ICG, respectively. Thus SLN detection rate with either ICG or Tc^99m^ was 98% (97/99 patients, 95% CI: 95.2–100%) (Table 2).

Histology revealed that 28 SLN (12.7%) from 21 patients (21.2%) had lymph node involvement (positive nodes). 23 of these positive nodes had been marked with both Tc^99m^ and ICG and 4 were positive for ICG fluorescence only, while 1 was only detected by gamma probing. Thus the detection rate for positive nodes by Tc^99m^ and ICG referring to the total amount of positive nodes was 85.7% (95% CI: 72.8–98.7%) and 96.4% (95% CI: 89.6–100%), respectively.

21 patients were node-positive (pN+). Using Tc^99m^ only, we would have missed 1 patient with node involvement, whereas ICG detected all pN+ patients. The reliability of detecting pN+ patients by using Tc^99m^ and ICG was 95.2% (95% CI: 86.1–100%) and 100% for ICG, respectively (Table 3). Hence, the false negative rates for Tc^99m^ and ICG were 4.8% and 0%, respectively.

## 4. Discussion

Detection rates of SLN in our study were 98% for ICG and Tc^99m^, respectively, and 100% for the combination. This is in the very high range when compared to results in the literature [4]. However, it is comparable with the detection rate achieved in the German-Austrian multicenter SENTINA trial. Here SLNB was reported to be successful in 1013 of 1022 patients who underwent SLNB before NAST, calculating a rate of 99.1%. This excellent result can be explained and valued as a consequence of a meticulous implementation process for SLNB which is described by Kuehn and colleagues [22] and ensured by a voluntary, external benchmarking programme and third-party dual certification of breast centers inaugurated and monitored by the German Cancer Society and the German Society of Senology [20, 23]. This certification demands defined experience of breast surgeons including SLNB procedure before they can take responsibility for surgical treatment of breast cancer patients and includes a mandatory quality management with annual external audits for all aspects of breast cancer diagnosis and treatment.

The true accuracy of SLNB using radiocolloid and/or ICG cannot be stated in our study because we did not perform routine axillary dissection as a control. The false negative rate of SLNB seems to be closely related to the number of sentinel nodes removed [23, 24]. In the prospective NSABP-B32 trial [24], a false negative rate of 9.8% was reported for all patients who underwent SLNB in primary surgery. However, women with only one detected sentinel node had a false negative rate of 17.7%, whereas those with at least two removed sentinel lymph nodes had a false negative rate of 10.0% or better. In our study, ICG discovered more SLN than did radiocolloid (215 of 220 nodes or 97.7% of all SLNB versus 172 nodes or 78.2%). This could be an indication for a possibly higher accuracy of ICG, or it might also indicate an excessive and unnecessarily more aggressive surgery due to ICG imaging. Nevertheless, it is important that Tc^99m^ missed one of 21 pN+ patients, while ICG recognized all 21, corresponding to false negative rates of 4.8% and 0%, respectively. Considering that this accuracy calculation has the limitation of missing true control (ALND), it is still within the range reported in thoroughly conducted clinical trials for Tc^99m^ [23, 24]. A false negative rate below 10% is considered acceptable for SLNB, and even axillary lymph node dissection (ALND) overlooks some pN+ patients [3, 23, 24].

The standard technique for axillary staging in cN0 patients is SLNB. German guidelines and certifications require that SLNB be used and the rate of axillary lymphadenectomy be kept very low in these patients [7, 8, 20]. Single-tracer labeling using Tc^99m^ is considered reliable and adequate and state-of-the-art [7, 8]. The accuracy of SLNB seems to be closely related to the number of sentinel nodes removed [23]. A dual-tracer method using Tc^99m^ together with blue dye has been shown to increase the number of SLN removed and improve detection and minimize false negative rates for SLNB. But it also increases the number of unaffected nodes removed from the axilla [11, 15, 23]. Thus, the advantage is not considered to be sufficient to justify the higher time and effort and the increased surgical trauma; therefore dual-tracer SLNB is not designated as standard of care but merely as optional [7, 8].

Our study using a combination of radiotracer and ICG as dye confirmed the benefits of dual tracer in improving detection rates and accuracy: SLNB was successful in 100% of patients and more tumor-affected lymph nodes were sampled than with any tracer alone but at the costs of removing more healthy nodes.

However, SLNB was not introduced to detect a large number of, or possibly all, axillary tumor-bearing nodes but solely to identify those cN0 patients that are actually pN+. Since all cN+ patients underwent consecutive axillary lymph node dissection (ALND) according to the original sentinel approach, the risk of leaving affected nodes in the axilla was negligible, even if SLNB did not remove them all. Therefore, it is of no concern if Tc^99m^ does not detect as many involved nodes (positivity rate of all tumor-affected nodes: 85.7%) as ICG (positivity rate: 96.4%), as long as it does not miss a pN+ patient.

On the other hand, an essential intention of the sentinel technique is to reduce the surgical trauma to the axilla and to minimize the removal of healthy lymph nodes. From this perspective, the higher number of unaffected nodes excised when using dual-tracer method is disadvantageous.

However, recent research has changed the need for ALND in all patients with tumor-bearing SLNB [6–8, 25]. Given that cN0 patients have planned lumpectomy, planned tangential whole-breast irradiation, and adequate adjuvant systemic therapy and no more than 2 involved SLN, leaving affected nodes in the axilla by not performing ALND apparently is not detrimental to 10-year disease-free survival and overall survival [25]. Since this statement applies only to no more than 2 affected nodes, the detection of tumors in more than 2 SLN changes the treatment in such a way that ALND becomes necessary. If a double-tracer SLNB can detect more tumor-bearing nodes than a single-tracer technique, this could increase the likelihood of revealing more than 2 involved nodes and therefore change the treatment strategy in certain patients. With today's knowledge, it is impossible to find out if this would be an advantage or a disadvantage for these patients.

Nodal status as a marker for guiding adjuvant treatment strategies decreases as tumor biology increases in weight [7, 8, 26]. Therefore, the unfavorable effect of not realizing the pN+ status in a cN0 patient dwindles, which reduces the potential danger of a false negative SLNB and further lessens the need for the dual-tracer SLNB.

When choosing the optimal tracer for SLNB, Tc^99m^ is the current favorite. However, the disadvantages of using radioactivity [9, 27], including the need for a nuclear medicine department with specific radiation protection and regulatory requirements and the extra time required for the injection of radiocolloid and lymphoscintigraphy, increase the desire for an alternative technique. Therefore whether ICG is inferior to radiocolloid is of interest.

Preoperative lymphoscintigraphy, which detects the number and position of SLN, is called an advantage of radiocolloid detection. However, the randomized, controlled, multicentric GBG 80-Senszi trial [28] proved that SLNB with radiocolloids is equally successful regardless of whether or not the surgeon is aware of the results of preoperative lymphoscintigraphy. Therefore, lymphoscintigraphy appears to be dispensable.

The use of NIR fluorescent dyes has many potential advantages such as relatively easy application, broader availability as compared to radiotracers, lower costs, and, real-time visualization, which facilitates the localization and excision of SLN. ICG is a low-cost pharmaceutical; time requirement is an excessive 30-minute waiting time between injection of ICG and start of surgery. The required additional technology for SLNB with ICG in our setup was just an exoscope and the fixture. Light fountain, HD NIR camera, and monitor can also be used for laparoscopic surgery.

In our experience, ICG is a reliable tracer for improving the accuracy of SLNB with Tc^99m^ colloid when used together. In contrast to Sugie and colleagues [19], we were unable to detect the subcutaneous lymphoid tracts in all cases, but this did not affect the detection rate and accuracy. Moreover, as a sole tracer used for SLNB, it is not inferior to the radiocolloid; in fact, the accuracy was better and the number of tumor-containing lymph nodes removed was higher. As discussed, it is unclear whether it is beneficial or rather harmful to define and remove more lymph nodes as SLN with ICG as a tracer.

A high accuracy of NIR fluorescence SLNB for nodal staging as demonstrated in our study has also been reported by others. Several studies and two meta-analyses confirmed the high concordance in SLN detection rate between ICG and Tc^99m^, ranging from 89% to 100% [17, 18, 27, 29–34]. ICG was also found to at least match the reliability of blue dye [19].

In a most recent meta-analysis, Sugie et al. [34] evaluated the diagnostic performance of the ICG fluorescence method compared with the radioisotope method. A total of 12 studies comprising 1736 women met their inclusion criteria. They did not find a significant difference between ICG fluorescence and radioisotope for SLN detection using different models. Concerning the detection rate for tumor-positive SLN, the ICG fluorescence method turned out to be significantly better than the radioisotope method for nodal staging in one but not all of their models used. They reported study outcome heterogeneity for the detection of SLN but not for tumor-positive SLN. The authors considered ICG to have valid diagnostic performance for SLN detection and reported a trend toward better axilla staging compared with the radionuclide method.

In another meta-analysis, Zhang et al. [30] also investigated the diagnostic performance of ICG-guided SLNB. Their inclusion criteria demanded complete ALND after SLN dissection regardless of the results of SLNB as gold standard for nodal staging. Nineteen studies comprising 2594 patients were included for assessing a pooled detection rate that was calculated to be 98%. Six studies including 254 patients with axillary dissection met the criteria for analysis of a pooled sensitivity and specificity. The authors calculated that ICG fluorescence-guided SLNB in breast cancer has a 98% detection rate and that the pooled sensitivity and specificity are relatively high at 92% and 100%, respectively. This meta-analysis required ALND to determine the false negative rate and calculated it to be 8%. This is comparable to the results of the NSABP B-32, in which the false negative rate for combined blue dye and radioisotopes was 9.8%. Another study that pooled data based on approximately 8000 patients reported the false negative rates at 10.9% for blue dye alone and 8.8% for radiocolloid alone, respectively [4]. A recent paper reviewed the evolution of SLNB as a useful tool in breast cancer management. The article reported that, in studies comparing SLNB and ALND, the false negative rate was mainly between 5% and 10% but it was even up to 16%. Nevertheless, the survival outcomes in these studies were the same in the SLNB and ALND groups and the frequency of axillary recurrences was below 1% after up to 10 years [35]. This confirms that the accuracy achieved with SLNB is considered acceptable [6, 23, 24].

Interesting to mention is the fact that the SLNB procedure using ICG for detection is also successful in patients who received neoadjuvant hormonal or chemotherapy [35, 36]. Accordingly, all SLN dissected from 19 patients of our study group who were treated with neoadjuvant therapy prior to the procedure were positive for ICG.

More controlled prospective studies are necessary to establish the standard use of ICG fluorescence for SLNB but more importantly to optimize its protocol. In particular, it was shown that the concentration of the ICG should be carefully selected. Most previous studies used a relatively high concentration (up to 6.4 mM) that could cause quenching of fluorophore, thus rendering the reabsorption of ICG-emitted photons undetectable [17, 37]. We used a 0.77 mM ICG solution, which is close to 0.5 mM, the concentration defined by Verbeek et al. to induce maximal NIR fluorescence [17].

Some research groups suspect a possible influence of a patient's BMI on the capacity of the ICG method based on the assumption that the fluorescence emission in lymph vessels and lymph nodes could be limited [29]. Consistent with this, the mean BMI of the 2 patients with SLN detected only by Tc^99m^ was 31.3, which is significantly different from the mean BMI of the entire study group (26.8, *p* < 0.001). Due to the small number of study participants, however, it is not possible to draw in-depth conclusions about the BMI influence.

Another aspect to consider is the surgeon's learning curve, which has an impact on the successful performance of the SLNB [38, 39]. One advantage of the ICG method over radioisotopes is the real-time visualization, which allows easy localization and simultaneous excision of the SLN. However, leakage of the ICG dye due to an incision of the lymphatic vessel leading to the SLN could cause the spread of fluorescence glow throughout the entire surgical field. To avoid this problem, we have tried to keep the lymphatic vessels as intact as possible by first spreading the tissue and not dissecting it. Others have suggested ligating the SLN main lymphatic duct [40].

In summary, our results indicate that ICG fluorescence is a valuable technique in addition to a radioactive tracer in performing SLNB in order to increase detection rate and accuracy. Furthermore, ICG has the potential to replace Tc^99m^ as a tracer for SLNB, with the advantage of lower costs and avoidance of radioactivity.

## Figures and Tables

**Table 1:** SLN mapping results.

	*N*	%
*Patients*	99	100,0

*Nodes detected per patient (mean, range)*	2.2	1–7
Zero	0	0,0
One	39	39,4
Two	29	29,3
Three	18	18,2
More than three	13	13,3

*Nodes detected*	220	100.0
Both tracers	167	75,9
By Tc^99m^	172	78,2
By Tc^99m^ only	5	2,3
By ICG	215	97,7
By ICG only	48	21,8

Patients with tumor-affected SLN	21	
*Tumor-affected SLN detected per patient (mean, range)*	1.3	1–3
Tumor-affected SLN	28	100.0
Tumor-affected SLN detected by both tracers	23	82.1
Tumor-affected SLN detected by Tc^99m^	24	85.7
Tumor-affected SLN detected by Tc^99m^ only	1	3.6
Tumor-affected SLN detected by ICG	27	96.4
Tumor-affected SLN detected by ICG only	4	14.3

**Table:** (a) Numbers and rates of nodes detected by Tc^99m^ and/or ICG

	Tc^99m^		
	Positive	Negative	Total
ICG			
Positive	167 (75.9%)	48 (21.8%)	215 (97.7%)
Negative	5 (2.3%)	0 (0.0%)	5 (2.3%)

Total	172 (78.2%)	48 (21.8%)	220 (100.0%)

**Table:** (b) Numbers and rates of patients with at least one node detected by Tc^99m^ and/or ICG

	Tc^99m^		
	Positive	Negative	Total
ICG			
Positive	95 (96.0%)	2 (2.0%)	97 (98.0%)
Negative	2 (2.0%)	0 (0,0%)	2 (2.0%)

Total	97 (98.0%)	2 (2.0%)	99 (100.0%)

**Table:** (c) Numbers and rates of tumor-affected SLN detected by Tc^99m^ and/or ICG

	Tc^99m^		
	Positive	Negative	Total
ICG			
Positive	23 (82.1%)	4 (14.3%)	27 (96.4%)
Negative	1 (3.6%)	0 (0,0%)	1 (3.6%)

Total	24 (85.7%)	4 (14.3%)	28 (100.0%)

**Table:** (d) Numbers and rates of patients with at least one tumor-affected SLN detected by Tc^99m^ and/or ICG

	Tc^99m^		
	Positive	Negative	Total
ICG			
Positive	20 (95.2%)	1 (4.8%)	21 (100.0%)
Negative	0 (0.0%)	0 (0.0%)	0 (0.0%)

Total	20 (95.2%)	1 (4.8%)	21 (100.0%)

**Table 3:** Detection rates for Tc^99m^ and ICG referring to different reference groups.

Reference group	Method	*N* SLN Detected/positive	*N* SLN Total	Detection rate (%)	Lower 95% CI (%)	Upper 95% CI (%)	False negative rate (%)
Total SLN	Tc^99m^	172	220	78,2	72,7	83,6	21,8
	ICG	215	220	97,7	95,8	99,7	2,3
All patients	Tc^99m^	97	99	98,0	95,2	100,0	2,0
	ICG	97	99	98,0	95,2	100,0	2,0
Tumor-affected SLN	Tc^99m^	24	28	85,7	72,8	98,7	14,3
	ICG	27	28	96,4	89,6	100,0	3,6
All pN+ patients	Tc^99m^	20	21	95,2	86,1	100,0	4,8
	ICG	21	21	100,0	—	—	0,0
